# Secure Surveillance of Antimicrobial Resistant Organism Colonization or Infection in Ontario Long Term Care Homes

**DOI:** 10.1371/journal.pone.0093285

**Published:** 2014-04-08

**Authors:** Khaled El Emam, Luk Arbuckle, Aleksander Essex, Saeed Samet, Benjamin Eze, Grant Middleton, David Buckeridge, Elizabeth Jonker, Ester Moher, Craig Earle

**Affiliations:** 1 Electronic Health Information Laboratory, Children's Hospital of Eastern Ontario Research Institute, Ottawa, Ontario, Canada; 2 Paediatrics, University of Ottawa, Ottawa, Ontario, Canada; 3 Electrical and Computer Engineering, Western University, London, Ontario, Canada; 4 Family Medicine, Memorial University of Newfoundland, St. John's, Newfoundland and Labrador, Canada; 5 Privacy Analytics Inc., Ottawa, Ontario, Canada; 6 Department of Epidemiology, Biostatistics and Occupational Health, McGill University, Montreal, Quebec, Canada; 7 Cancer Research Program, Institute for Clinical Evaluative Sciences, Toronto, Ontario, Canada; Peking Union Medical College, China

## Abstract

**Background:**

There is stigma attached to the identification of residents carrying antimicrobial resistant organisms (ARO) in long term care homes, yet there is a need to collect data about their prevalence for public health surveillance and intervention purposes.

**Objective:**

We conducted a point prevalence study to assess ARO rates in long term care homes in Ontario using a secure data collection system.

**Methods:**

All long term care homes in the province were asked to provide colonization or infection counts for methicillin-resistant *Staphylococcus aureus* (MRSA), vancomycin-resistant enterococci (VRE), and extended-spectrum beta-lactamase (ESBL) as recorded in their electronic medical records, and the number of current residents. Data was collected online during the October-November 2011 period using a Paillier cryptosystem that allows computation on encrypted data.

**Results:**

A provably secure data collection system was implemented. Overall, 82% of the homes in the province responded. MRSA was the most frequent ARO identified at 3 cases per 100 residents, followed by ESBL at 0.83 per 100 residents, and VRE at 0.56 per 100 residents. The microbiological findings and their distribution were consistent with available provincial laboratory data reporting test results for AROs in hospitals.

**Conclusions:**

We describe an ARO point prevalence study which demonstrated the feasibility of collecting data from long term care homes securely across the province and providing strong privacy and confidentiality assurances, while obtaining high response rates.

## Introduction

Antimicrobial resistant organisms (AROs), such as methicillin-resistant *Staphylococcus aureus* (MRSA), vancomycin-resistant enterococci (VRE), and extended-spectrum beta-lactamase (ESBL)-producing micro-organisms, cause considerable morbidity and mortality, and are, by their nature, difficult to treat and can cause hospitalization and reduction in overall health status of long term care home (LTCH) residents [1]. In addition to the serious implications to patients, attempts to contain ARO also require extra resources within healthcare facilities, through the use of additional precautions, cohorting of patients, single patient rooms, and increasing or specialized housekeeping practices.

A number of studies of the prevalence of AROs in long-term care have been conducted in the United States and in Europe using laboratory confirmation of the micro-organism as the outcome measure [2]–[11]. These studies have shown high variability in the proportion of residents of long-term care facilities who are colonized or infected with AROs, with estimates reaching as high as 74.8% of residents found to be carrying at least one ARO [4], and the carriage rate of MRSA varying from 11% to over 48% of residents in LTCHs [7]–[11]. A prevalence study of known MRSA cases in US healthcare facilities was undertaken in 2006, and relied on patient medical records to determine known MRSA-case status. This was the first ever nation-wide prevalence study of MRSA in the US, which had participation from 1237 facilities, representing 8654 MRSA colonized or infected patients [3].

Rates of MRSA and VRE in healthcare settings have been rising in Ontario since 1996 [12]. It is estimated that the majority of ARO's are acquired in the hospital or community setting (41% and 42% respectively), rather than LTC settings (17%) [12]. However, opportunities for inter-facility transmission of ARO are ubiquitous as patients and residents are transferred between acute and long-term care settings.

Hospital-based surveillance of some of these organisms is mandated in Ontario using accepted definitions and systematic data collection protocols and specific results, such as rates of MRSA and VRE bacteremia, and are reported publicly as patient safety indicators [13]. However, the burden of ARO in long term care settings in Ontario has not been measured to date [14], [15]. There is no current requirement to report ARO colonization and infection rates to the public or to public health authorities. Consequently, at the time of the study reported on in this paper, there was no ARO colonization or infection baseline for LTCH's in Ontario. When surveillance data is unavailable, it can be difficult to make informed decisions and to identify needs.

There is stigma attached to the identification of residents carrying an ARO, characterized by higher insurance payments or denial of insurance coverage [16], refusal of facilities to accept such patients [2], significantly longer placement delays [17], and recommended universal gloving when in contact with such residents [18]. Given that almost 60%of LTCHs are privately owned [15] and that home funding consists of a mixture of government support and monthly resident fees, this may also increase resistance to sharing ARO data and having that information become known publicly for fear that it may affect funding or deter transfers and potential new residents. It has been argued that in commercial settings, or where there may be political consequences for reporting performance data, there is reluctance by public health surveillance data sources to share information [19]–[22]. A recent study demonstrated resistance by Canadian providers to share data with public health due to patient privacy and performance evaluation concerns [23]. There is also evidence that Canadian healthcare facilities are reluctant to share data on their infection rates as well as on other performance indicators, and this reluctance is re-enforced by provincial and federal health authorities [24]. To address such data sharing concerns for public health surveillance programs, in a previous study we had developed a theoretical secure protocol for the computation of rates without revealing the values from individual sites providing the data and that also protects the identity of residents [25]. This protocol addresses the stigma and the data sharing concerns noted above.

In this paper we describe how we customized and extended this protocol, applied it to a real-world provincial surveillance program to establish baseline ARO colonization and infection rates for LTCHs, and identified the practical implementation issues that had to be addressed in deploying a secure surveillance system.

Both colonization and infection rates were collected. Colonization is usually asymptomatic and would only be detected through screening. Individuals who are colonized may progress to an infection, and do pose a risk of transmission to others.

The contributions of this work are: (a) the development and deployment of a provably secure ARO surveillance system, (b) the system provides anonymous feedback directly to the homes to allow for internal and external benchmarking which is considered important for improving practices [15], and (c) reporting on the first province-wide evaluation of ARO prevalence in Ontario LTCHs.

## Methods

### Requirements

The objective of our study was to develop and use a secure surveillance system to establish the prevalence of AROs in Ontario LTCHs. The secure surveillance system had to meet the following requirements:

1. Compute mean colonization or infection rates and their standard deviations by region and facility size.
2. The probability of re-identifying individual residents with an ARO colonization or infection should be very small.
3. The probability of determining the colonization or infection rate for any particular home should be very small.

We examine how these requirements were met in the ‘Methods” section of the paper. Note that requirement R3 is not to hide the identity of the homes, but only to not reveal their actual colonization or infection rates (we needed to know their identity to follow-up, send reminders, and send them their benchmark results).

### Recruitment

Our objective was to conduct an assessment of the number of known cases of MRSA, VRE and ESBL in LTCHs across the province. Data collection was conducted in the Fall of 2011. The Regional Infection Control Networks (RICN) at Public Health Ontario (PHO) invited all 621 LTCHs to participate via an email letter sent to them by their respective RICN coordinator. Reminder emails and telephone calls were targeted at LTCHs that had not yet submitted data throughout the month-long period in which LTCHs were able to submit data.

### Data Collection

Previous studies indicated that approximately 70% of Canadian LTCHs conduct admission screening for some AROs [26]. Regulations under the Long Term Care Homes Act, 2007, require homes to monitor, record, and analyze information daily relating to the presence of infections in residents [15]. Therefore data on post-admission acquisition of an ARO should also be available.

The planned data collection period was October 17, 2011 to November 18, 2011. The LTCH Directors of Care, or designate, were asked to select one day during the study period to enter their data through a secure web-based portal. The following data were requested: a) the number of residents in the LTCH on the date of data submission and, b) the number of known VRE, MRSA and/or ESBL positive residents on the date of data submission. Residents colonized or infected with the micro-organisms of interest were identified only by their known status at the time of the survey (i.e., through their medical records); no screening or laboratory-testing was conducted as part of this project. Residents who were known to be colonized or infected with more than one ARO were counted in each relevant category.

### Data Collection and Management

**Figure 1:**
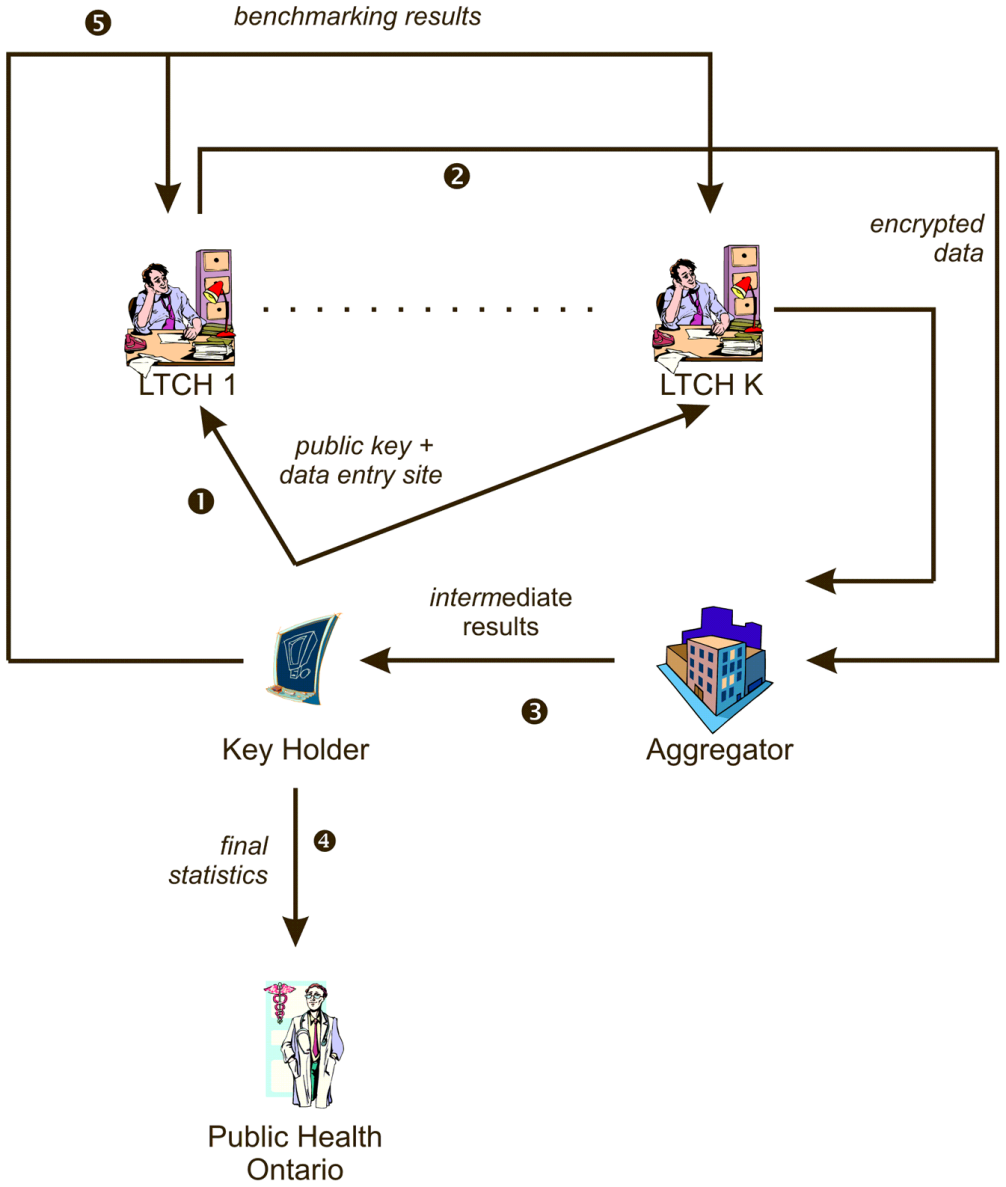
The flow of information in the secure surveillance system.

The secure protocol and system for collecting the data is shown in Figure 1. We assumed that there were *K* LTCHs. The data collected from the LTCHs and the resultant statistics computed from that data were sent to PHO. Two semi-trusted third parties participated in this protocol: a Key Holder (KH) and an Aggregator. The KH was the Children's Hospital of Eastern Ontario Research Institute in Ontario and the Aggregator was McGill University in Quebec. A definition of semi-trusted third parties and a comparison to alternative types of third parties that can be used in secure computation protocols is provided in Appendix S1.

The protocol used a special type of cryptosystem that allows one to perform mathematical operations on the encrypted data, called the Paillier cryptosystem [27]. Paillier utilizes a public-private key pair to encrypt and decrypt the data. This is described briefly in Appendix S1. In our case the mathematical operations were combined to compute means and standard deviations. The details of these secure computations are described in more detail in Appendix S1.

The five steps in the protocol were as follows:

1. The KH generated a key pair, and sent the public key to the Aggregator. The user at each LTCH navigated to a web page with a data entry form. Embedded within the web page was the public key. Each LTCH user then entered their four count values in this on-line form.
2. When the user submitted their data, a JavaScript implementation of Paillier within the web browser encrypted the values using the public key, and then these encrypted values were transmitted to the Aggregator. In addition to the encrypted values, the Aggregator stored a timestamp and the ID of the submitting LTCH. When an LTCH submitted its data, the Aggregator informed the KH of the ID of the responding LTCH.
  Based on our performance testing, we found that there was considerable variation in the average encryption time of a single value across web browsers (see Table 1). Older versions of Internet Explorer were quite slow and this would have frustrated the users. Users of Internet Explorer 8 or lower were therefore given the option to install Chrome Frame, which is a quick-to-install plugin for Internet Explorer that embeds the much faster JavaScript engine of the Chrome browser.
  The Aggregator stored all of the incoming encrypted data. The browser at each site also stored the submitted values locally within a browser (Flash) cache at the LTCH.
3. At the end of the data collection period, the Aggregator computed the desired statistics on the encrypted data collected thus far: average colonization or infection rates per 100 residents by region and bed size of the facility, and their standard deviations. The exact computations performed by the Aggregator are described in further detail in Appendix S1. The encrypted results of the above computations were then sent to the KH.
4. The KH decrypted the computed statistics that it received using its private key, and sent these results to PHO. These statistics allow us to meet requirement R1.
5. The LTCHs were able to access a web site at the KH to benchmark their own results against other homes of the same size and within the same region. The KH sends the aggregate results to the LTCHs and these were compared to the cached results (in the local browser) that the LTCH originally submitted.

**Table 1:** Time to encrypt an integer using JavaScript in different browsers.

Browser and Version	Time to Encrypt a Single Integer (seconds)
Internet Explorer 8	79.97
Internet Explorer 9	17.54
FireFox 9.0.1	21.17
Chrome 16	2.1
Safari	48

The timing data was collected as an average of 100 integers on a Windows 7 virtual machine with a dual 3.2 GHz processor and 3 GB of RAM.

Because the protocol does not collect any information about the individual cases, it is not possible to determine the identity of individual residents from the data. Hence requirement R2 is met. If demographics, for example, were being collected about the cases then standard de-identification techniques could be applied [28].

During the data collection period, non-responding LTCH's were sent reminders by their RICNs. It was possible to identify non-respondents since the ID of respondents was recorded.

The LTCHs were categorized by size (1–60 beds, 61–120 beds, 121–180 beds and over 180 beds) and by geography (West, Central West, Toronto, Central East, East and North), using the boundaries identified for Local Health Integration Networks (LHIN) in Ontario. These boundaries were meaningful to local public health units as they represent geographies where they have authority to intervene. This resulted in a 4×5 table.

The Aggregator only receives encrypted data from the LTCH's. Therefore, there is negligible risk that the Aggregator would be able to determine the colonization or infection rate for any particular LTCH. The computed statistics, and not the encrypted data received from the LTCH's, are sent to the KH as noted in step 3 above.

The KH is able to decrypt values it receives. We wanted to ensure that the KH cannot use the values that it receives (and decrypts) as part of the protocol to reverse engineer the rate for any single LTCH. The specific bound we defined was that an attempt to reverse-engineer a home's rate would give at least five plausible values (so the probability of guessing the correct rate is at most 0.2). We termed this 5-identifiability. The probability of 5-identifiability has to be less than or equal to 0.05.

For example, the probability of 5-identifiability for any cell in the bed size by region results table should be less than or equal to 0.05. The same bounds must apply to marginal cells in the same table. To achieve that objective, the minimum number of reporting homes in each cell had to meet the constraints in Table 2. For instance, for homes with up to 60 residents, at least 12 homes have to submit valid data in each cell to allow the reporting of results for that cell. We describe in Appendix S1 how these boundary values were computed.

**Table 2:** Number of sites needed to ensure *Pr*(5-identifiability) ≤0.05 for a 10% colonization rate.

Facility Size	Number of Facilities
1–60	12
61–120	7
121–180	6
181–240	6

In cases where data was collected from fewer than the number of homes in a cell according to Table 2, then values were not computed by the Aggregator, and the Aggregator did not transfer that information to the KH. With such constraints in place, we were able to ensure to the homes that the probability of reverse engineering their rates was very small, and hence meet requirement R3.

### Validation

Since we did not actually access the raw data, how can we know that the computed rates are the correct values? There are two considerations. The first is the accuracy of the protocol itself. The derivation of the computations used in protocol itself are included in Appendix S1 and can be shown to be correct. The second concern is the software implementation. To address this concern the software was tested extensively with fake data sets before being deployed on the full surveillance project. The test cases chosen were intended to exercise boundary conditions of no, extremely high, and extremely low counts, as well as counts that are impossible (e.g., colonization or infection numbers that are greater than the total number of residents in the home). Through this exercise we gained additional assurance that the values computed on the encrypted data using our software are indeed correct.

### Data Analysis

Prevalence of ARO was calculated for each stratum, region and facility size, using the total number of cases in that stratum divided by the total number of residents in that stratum, multiplied by a constant of 100. Standard deviation from the overall prevalence was calculated for each region.

For each micro-organism, the chi-square test was used to test the difference in prevalence of the various bed groups against the overall prevalence, as well as for the difference in prevalence of the various regions against the overall prevalence.

### Non-response Bias

A common way to test for non-response bias is to compare early responders with late responders, where the late responders are treated as a proxy for non-respondents [29], [30]. In essence the respondents are divided into two groups and these are then compared on their colonization or infection rates. We considered those responding after the first 4 weeks of data collection as late respondents (representing 25% of all responding homes). To check for non-response bias we used a randomization test.

When dealing with data that is not from a random sample of a population, as is the case here, it is worth considering alternative statistical measures than those from classical statistics. In the case of testing for a difference between means of two groups, the classical approach would be to use a t-test or F-test. A randomization test, however, does not require that we have a random sample of a population, yet still allows us to make inferences regarding the data. Further, if we did assume random sampling then both the two-sample t-test and randomization test are valid under the same assumptions [31], [32].

A two-sample randomization test, or randomized t-test, requires that we randomize the data into two groups of the same size as the original groups. For each randomization the mean difference between groups is calculated. The randomizations create a frequency distribution that can be used to test the null hypothesis that there is no difference between groups. No assumptions are made, or required, with regards to a population distribution (unlike a t-test which assumes a normal distribution within groups, under a population model).

In addition, a randomization test is simple to implement within the context of the secure protocol. The Aggregator performs Monte Carlo randomizations and each time computes the statistic of interest. The results of all of these iterations are sent to the KH, which decrypts the statistics and computes the empirical p values.

### Ethics

This study was approved by the ethics board of the Children's Hospital of Eastern Ontario Research Institute. Only home-level counts were collected rather than individual resident-level data. The ethics board did not request individual resident consent since the data collected would be considered anonymized, and hence can be collected without consent. The representative of each home was invited to participate in the study through the letter that was sent to them, and them actually going to the web site and submitting data was considered as clear evidence of consent.

### Availability of Study Data

Data collected from the LTCH's was encrypted. To decrypt the original data collected from the LTCH's would violate the security of the protocol as it would require collusion among the semi-trusted parties involved, would violate the assurances provided to the Research Ethics Board that approved the study, as well as violate the statements provided to the LTCH's that participated in the study. Therefore, the data available from the first author, upon request, consists of the computed statistics provided by the Aggregator.

## Results

Of 621 LTCHs in Ontario, 515 (82.9%) responded. Data were eligible for aggregation for 512 (82.4%) as summarized in Table 3. Data representing 64,082 LTCH residents were collected.

**Table 3:** Number of responding facilities (n = 512) and total facilities (n = 621) by region and bed-group.

Region	1–60 Facilities participating/total	61–120 Facilities participating/total	121–180 Facilities participating/total	180+Facilities participating/total	Facilities participating/total (% participating)
North	15/18	19/25	10/15	5/5*	49/63 (77.7)
East	23/23	34/34	25/25	13/15	95/97 (97.9)
Central East	16/16	35/41	43/49	30/34	124/140 (88.6)
Toronto	3/6*	5/7**	10/12	12/13	27/38 (71.1)
Central West	12/13	40/46	51/56	19/22	122/137 (89.1)
West	23/34	42/68	23/34	7/10	95/146 (65.1)
**Total (% participating)**	**89/110 (80.9)**	**175/221 (79.2)**	**162/191 (84.8)**	**86/99 (86.7)**	**512/621 (82.4)**

* These cells did not meet the minimum cell size requirements by definition (there were insufficient homes to start off with), therefore these number could not be reported at the cell size.

But they can be included in the marginal values. This is why these cells are missing in the results tables.

** This cell did have sufficient homes but too few submitted data and therefore its values cannot be reported.

Our non-response bias results indicate that there was a statistically significant difference between respondents and non-respondents in terms of their VRE rates (p = 0.0164) and MRSA (p = 0.0024), while there was no difference for ESBL (p = 0.508). Non-respondents had higher colonization rates than respondents. This suggests that the values that are presented here may be undercounting known colonization rates in the province, and should be seen as a floor.

With 1920 cases of MRSA identified, the overall prevalence of MRSA was 3.0 cases per 100 residents. For VRE, 358 identified cases gave an overall prevalence of 0.56 cases per 100 residents, and the 523 cases of ESBL resulted in an overall prevalence of ESBL of 0.82 cases per 100 residents (see Table 4). Regional prevalence of MRSA ranged from 0.79 to 8.04 cases per 100 residents as summarized in Table 5. For VRE, the prevalence ranged by region from 0.13 to 1.28 cases per 100 residents (see Table 6). For ESBL, the range was 0.22 to 1.59 cases per 100 residents (see Table 7). The West region had the highest prevalence of MRSA and VRE compared to other regions in the province, whereas ESBL cases were identified with greater frequency in Toronto and the West region.

**Table 4:** Overall prevalence of AROs as reported by LTCH.

ARO	Cases	Prevalence (per 100 residents)
MRSA	1920	3.0
VRE	358	0.56
ESBL	523	0.82

**Table 5:** Prevalence of MRSA cases per 100 residents by region and facility size.

	Regions							
Facility number of beds	*North*	*East*	*Central East*	*Toronto*	*Central West*	*West*	Bed group prevalence	SD
1–60	1.57	3.17	0.72	–	3.31	8.38	3.87	3.24
61–120	1.07	2.04	1.8	–	2.73	7.88	3.34	2.85
121–180	0.56	2.54	1.08	0.91	3.15	7.83	2.94	2.58
180+	–	2.37	1.68	2.58	2.91	8.63	2.61	2.1
**Regional prevalence**	0.79	2.42	1.44	1.86	3.00	8.04		
**SD**	0.46	0.38	0.42	1.15	0.22	0.37		

**Table 6:** Prevalence of VRE cases per 100 residents by region and facility size.

	Regions							
Facility number of beds	*North*	*East*	*Central East*	*Toronto*	*Central West*	*West*	Bed group prevalence	SD
1–60	0	0.5	0	–	0	1.76	0.61	0.82
61–120	0.18	0.92	0.1	–	0.52	1.1	0.61	0.43
121–180	0.28	1.4	0.11	0.77	0.22	1.35	0.58	0.62
180+	–	1.09	0.42	0.2	0.28	1.21	0.49	0.42
**Regional prevalence**	0.13	1.08	0.23	0.38	0.29	1.28		
**SD**	0.14	0.33	0.19	0.33	0.16	0.24		

**Table 7:** Prevalence of ESBL cases per 100 residents by region and facility size.

	Regions							
Facility number of beds	*North*	*East*	*Central East*	*Toronto*	*Central West*	*West*	Bed group prevalence	SD
1–60	1.57	0.67	2.16	–	0.18	4.06	1.91	1.62
61–120	0.6	0.16	0.55	–	0.29	1.43	0.69	0.59
121–180	0.77	0.25	0.64	1.81	0.77	0.36	0.66	0.4
180+	–	0.06	1.47	1.44	0.33	0.51	0.87	0.67
**Regional prevalence**	0.73	0.22	1.04	1.59	0.52	1.19		
**SD**	0.37	0.2	0.6	0.25	0.29	1.36		

Prevalence by bed group was not significantly different from the overall prevalence for VRE, but there were some significant differences by bed group for MRSA and ESBL. Facilities in the 1–60 and 61–120 bed groups had significantly higher prevalence of MRSA than the overall prevalence (p = 0.001 and 0.026, respectively), whereas facilities in the 180+ bed group had significantly lower prevalence of MRSA than the overall prevalence (p = 0.004). Facilities in the 1–60 bed group had significantly higher prevalence of ESBL (p<0.001) than the overall prevalence, whereas facilities in the 121–160 and 180+ bed groups had significantly lower prevalence than the overall prevalence (p = 0.02).

## Discussion

### Summary

This was the first province-wide assessment of ARO prevalence in LTCHs in Ontario, with benchmarking feedback to facilities allowing them to compare their prevalence with facilities of the same size and in similar geographic area. This study found that overall, MRSA was the micro-organism most frequently identified, at 3.0 cases per 100 residents, with higher prevalence in the West region. This is followed by ESBL at 0.82 cases per 100 residents and VRE with 0.56 cases per 100 residents. The known presence of VRE and ESBL organisms across all geographic areas remains relatively small compared to MRSA.

Given that MRSA is the most prevalent ARO in Ontario [12], it is not surprising that it is also the most prevalent ARO in LTCHs. Similarly, our finding of higher MRSA and VRE prevalence in the West region is consistent with provincial laboratory data for hospital-based and community-based laboratories providing testing services to hospitals [12]. The prevalence of ESBL was highest in Toronto, almost double that of the overall prevalence of ESBL. This phenomenon may be explained by greater awareness, perhaps resulting in increased screening, arising from a large ESBL outbreak that was identified in Toronto in 2001 [26].

An unexpected finding of this project was that larger facilities tended to have fewer known cases of all three microorganisms. This may be a product of having a higher number of residents in the facility, which dilutes the number of known cases, resulting in a lower prevalence. Still, it remains unclear why larger facilities would not have proportionally more cases relative to their size. Given our intentional collection of a minimum data set, it was not possible to examine the factors that may explain this lower prevalence.

One driver for using a secure protocol was to provide strong and provable guarantees about the protection of the identity of the residents and ensure that values from individual homes could not be revealed. While the overall response rate was quite high, we did find that homes with higher rates of MRSA and VRE were less likely to respond. We have generated a number of hypotheses to explain this finding and these will be tested in future work [33].

### Benefits of a Secure Protocol

In this study we mentioned the use of the secure protocol and platform in communications with the LTCHs and the information letter we sent to them. One may expect that homes with high colonization rates may be concerned that relatives would pull residents out, or those with very low rates may be accused of doing insufficient testing. In both cases continued government funding may be affected. Therefore, a secure protocol would convince them to respond. However, such an expectation would be inconsistent with the literature. Existing behavioral economics literature would suggest that emphasizing the secure data collection system would have resulted in a *lower* response rate, especially where the sensitivity of the information collected is highest.

Conchie and Burns found that open communication with employees about occupational risks within high-risk organizations was found to increase trust beliefs; however, such information affected workers' trust *intentions* in a different manner [34]. Trust intentions are one's intentions to, or the likelihood of, one acting in a trusting (or distrusting) manner. Given information about an increase in risk, they found workers' trust intentions were reduced “irrespective of their existing levels of trust in risk management” [34]. Therefore, given information about risk, workers expressed intentions to behave in a less trusting manner. In our context this would likely translate into a reduction of the response rate when the risk is perceived to be high – which was consistent with what we found.

Directing our attention to online privacy behaviour, we find similar trends in how risk information influences behaviour. Presenting information about privacy can “prime” the user to be less trusting and more reluctant to complete transactions or disclose personal information online [35], [36].The salience of the privacy information worked to increase individuals' privacy concerns and decrease their willingness to disclose personal (sensitive) information.

Therefore, one can hypothesize that by implementing a secure data collection protocol and informing the homes about it, they were primed to think about how this data may be used. Such priming inhibited the willingness of homes with the highest colonization or infection rates from responding. It should be cautioned that this is a preliminary conclusion and would need to be further verified in a focused study (e.g., with a control group that did not receive any communication about the secure protocol in use) [33].

Perhaps the primary benefit from a secure protocol is that it constituted a key part of the case made to expedite the REB approval of the protocol. In our project we were able to start data collection within two weeks of PHO deciding to perform a point prevalence study.

### Limitations

There are several limitations to this study. First, we identified only known cases without the use of a laboratory-based screening protocol. Therefore, only cases identified previously through screening or other indication for testing are included. Known prevalence rates are heavily dependent on the intensity of ARO screening, which was not directly assessed. Our study was similar to the first ever nation-wide prevalence study of MRSA in the US undertaken in 2006, and which also relied on patient medical records to determine known MRSA-case status [3]. Other studies of known cases at the state level also used surveys of the facilities [2], [5], [6].

Data quality is greatly affected by the medical records from which the known cases were identified. LTCHs with more rigorous screening programs and more resources dedicated to infection prevention and control may identify more cases than those that lack a systematic and thorough approach to screening. Therefore, the known prevalence found in this study likely represents an underestimate of ARO carriage in LTCHs in Ontario.

The value of the study, however, is that the “known” cases in LTCH's represent the burden of AROs on the long-term care system, requiring additional resources, in terms of nursing time, additional transmission-based precautions, cohorting of patients, repeat screening, and so on. Furthermore, a province-wide study of “known” cases has the potential to capture data that reflects over 77,000 LTCH residents in the province, a scope which is far greater than would be feasible if using a methodology that required screening and laboratory-confirmation of all patients.

It may be possible under certain conditions to infer the values for some homes. For example, if all sites but one decide to publish their data, then the remaining site's rates would be known. Also, in cases where the variation is very small it would be known that all sites in that stratum are close to the published rate. This can be easily mitigated by not publishing exact rates when the standard deviation is low (instead, for example, publish a range or that the value is “less than” a number).

## Conclusions

We have demonstrated the feasibility of carrying out a microbial surveillance study across the province with timely, secure methods that can protect the values provided by individual respondents, but still allow the computation of relevant statistics. Strong security proofs for the protocol were also provided.

## Supporting Information

Appendix S1 **Trust, Ethics, and Detailed Calculations.**
(PDF)
