# Supplementary material for: Secure Surveillance of Antimicrobial Resistant Organism Colonization or Infection in Ontario Long Term Care Homes
Source: PLoS One. 2014 Apr 8;9(4):e93285. doi: 10.1371/journal.pone.0093285 (PMC3979675; doi:10.1371/journal.pone.0093285)
Supplement: Appendix S1 — Trust, Ethics, and Detailed Calculations. (PDF) [file pone.0093285.s001.pdf]

Appendix S1: Trust, Ethics and Detailed Calculations

Trust and Third Parties

In secure computation protocols third parties are often necessary. These parties receive data, perform some computation on that data, and then forward the results of that computation. There are three types of third parties: (a) malicious, (b) trusted, and (c) semi-trusted.

A malicious third party would deliberately attempt to attack the data it receives to recover personal or sensitive information. There is no guarantee that this party would even follow the protocol and may inject false data in the output stream. Protecting against a malicious third party results in protocols that are exceedingly complex and would their performance not be practical for real-life deployment. In healthcare settings this assumption is not usually made [1–3].

A trusted third party can receive raw data that is sensitive and identifiable. There are three challenges with having a trusted third party in a protocol: (a) disclosures if the third party is compromised or corrupted, (b)compelled disclosures, and (c) all LTCH`s must trust the third party.

The first challenge is that if a trusted third party`s security is compromised, the adversary will have access to the raw data of the LTCH`s and their corresponding counts. A compromise can be due to either insiders or outsiders. A compromise can be as simple as a change your password phishing attack to obtain the credentials of an employee of the third party. Many social-engineering techniques exist[4,5],and have been used to obtain passwords and very personal information from individuals and organizations (as well as to commit more dramatic crimes such as bank robberies)[6,7].A recent review of data breaches indicated that12% of data breach incidents involved deceit and social-engineering techniques[8].Corruption can occur if an individual with access to the raw data within the trusted third party is bribed or blackmailed to reveal information.

Second, a trusted third party could be compelled to disclose personal health information, for example, in the context of litigation. For research in the US, the National Institutes of Health can issue certificates of confidentiality to protect identifiable participant information from compelled disclosure, and allow researchers to refuse to disclose identifying information in any civil, criminal, administrative, legislative or other proceeding, whether at the federal, state or local level[9].However, these would not be applicable to non-research projects or to projects that are not approved by an IRB, and most public-health surveillance programs would be in that excluded category. Furthermore, there are mixed views on the effectiveness of certificates of confidentiality [10–13], and such certificates do not exist outside the USA.

Third, the trusted third party must be trusted by all of the LTCHs supplying data. This creates potential obstacles to the release of that data and may result in a long start-up and negotiation time for surveillance efforts and strong non-response bias.

To address these challenges, we propose a distributed protocol with the weaker requirement of having only semi-trusted third parties. A semi-trusted third party would not be able to access any of the raw data, even if it wanted to. This means that if there is a security compromise, staff corruption, or a compelled disclosure, there is no additional risk of viewing raw data. A protocol with semi-trusted third parties also overcomes the requirement of practices having to completely trust the third party. The only requirement on a semi-trusted third party is that it follow the protocol faithfully.

Ethics Considerations

The use of secure surveillance system should expedite the ethics review process because there are no major privacy issues to contend with. In addition, because no information that would identify residents, or that would implicate LTCHs, was being collected, we examined whether this kind of study required ethics review at all.

In Canada, REBs follow the Tri-Council Policy Statement: Ethical Conduct for Research Involving Humans (TCPS) [14]. Article 2.4 of the TCPS states that no REB review is required if a research project relies on the secondary use of "anonymous" data. The document goes on to explain that anonymous information and human biological materials are distinct from those that have been coded, or anonymized. Whereas anonymous data never contained identifiers, anonymized data was previously identifiable but later irreversibly stripped of this information.

In this study we collected existing colonization and infection data from the medical records of LTCH residents. The secure protocol ensures that the identity of residents cannot be determined and that individual facility values cannot be computed. This de-identification process is irreversible. However, the mere existence of a private key that can decrypt the data, even if the protocol uses multiple parties to ensure that does not happen, means that the data cannot be anonymous as defined in the TCPS.[1] Also, that the data entered by the LTCHs into our system existed originally in identifiable form in electronic medical records also means that the data collected is not anonymous (because there exists an identifiable version of that data), even if this identifiable data is not collected for the study.[2] Therefore, according to that reasoning we were collecting anonymized data, which is not exempt from ethics review.

Moreover, even if data is anonymous (or anonymized), some have argued that individuals may have a property interest in their personal information, suggesting the need to consult with affected individuals in some manner [15], although this is not a generally accepted legal theory. On the other hand, it can also be argued that studies with anonymous (or perhaps, anonymized) data can still present ethical risks due to the possibility of group harm [16]. For example, a research protocol restricted to the study of anonymized information might nevertheless, classify residents on the basis of their ethnicity or race (e.g., those of Aboriginal origin versus those not of Aboriginal origin). It may therefore be possible to draw conclusions about Aboriginal communities on the basis of these research data. These inferences could be harmful to Aboriginals as a group; namely, in underscoring social stigma or associating a

---

[1]TCPS interpretation team, Panel on Research Ethics, personal communication.
[2]TCPS interpretation team, Panel on Research Ethics, personal communication.

group with higher prevalence of infections or colonizations. An REB would be in a position to make that determination.

Therefore, the definitions in Article 2.4 *still require* secure data collection protocols to go through ethics review, as well as the more general need to examine the potential for group harm. We presented this protocol to the Children's Hospital of Eastern Ontario Research Institute REB. The use of a secure protocol expedited the review process considerably since there were little privacy risks or risks of inadvertent harm to the homes, and there were no evident group harms to consider.

Paillier Cryptosystem

We use the additive homomorphic encryption system proposed by Paillier [17] for our secure computations. With the Paillier cryptosystem it is possible to perform mathematical operations on the encrypted values themselves, such as addition and limited forms of multiplication. Formally, for any two data elements m_1 and m_2 and their encrypted values, $E(m_1)$ and $E(m_2)$, the following equation is satisfied:

$$D\left(E(m_1)\times E(m_2) \bmod p^2\right) = m_1 + m_2 \bmod p \qquad \text{..................... (1)}$$

where p is a product of two large prime numbers, and D is the decryption function. In this type of cryptosystem addition of the plaintext is mapped to the multiplication of the corresponding ciphertext. The Paillier cryptosystem also allows a limited form of the product of an encrypted value:

$$D\left(E(m_1)^{m_2} \bmod p^2\right) = m_1 \times m_2 \bmod p \qquad \text{..................... (2)}$$

which allows an encrypted value to be multiplied with a plaintext value to obtain their product.

Another property of Paillier encryption is that it is probabilistic. This means that it uses randomness in its encryption algorithm so that when encrypting the same message several times it will, in general, yield different ciphertexts. This property is important to ensure that an adversary would not be able to compare an encrypted message to all possible counts from zero onwards and determine what the encrypted value is.

Depending on the context we also use the simpler notation where we let $[\![m]\!]$ denote the encryption of m under a semantically secure additively homomorphic public-key encryption algorithm, such as Paillier [17].

Weighted Sample Mean and Variance

We summarized the LTCH's into a two dimensional contingency table, with the number of beds (rows) by the region (columns). The colonization rate per 100 residents in any facility in that table is given by:

$$r_{ijk} = \frac{c_{ijk}}{n_{ijk}} \times 100,$$

for row i, column j, and facility k, where c_{ijk} is the number of cases and n_{ijk} is the number of residents at the facility.

Weighted Mean

The weighted mean (by number of residents) for a cell is given by:

$$m_{ij} = \frac{\sum_k n_{ijk} r_{ijk}}{\sum_k n_{ijk}}.$$

The weighted mean for cell (i, j) can be rewritten as:

$$m_{ij} = \frac{\sum_k n_{ijk} r_{ijk}}{\sum_k n_{ijk}} = \frac{\sum_k n_{ijk} \left(\frac{c_{ijk}}{n_{ijk}} \times 100 \right)}{\sum_k n_{ijk}} = \frac{\sum_k c_{ijk}}{\sum_k n_{ijk}} \times 100$$

Therefore, the weighted cell mean by number of residents is also the pooled rate of colonizations.

The weighted (marginal) mean for row i is given by:

$$m_{i+} = \frac{\sum_j n_{ij} m_{ij}}{\sum_j n_{ij}} = \frac{\sum_j \left(\sum_k n_{ijk} \right) \left(\frac{\sum_k c_{ijk}}{\sum_k n_{ijk}} \times 100 \right)}{\sum_j \left(\sum_k n_{ijk} \right)} = \frac{\sum_j \sum_k c_{ijk}}{\sum_j \sum_k n_{ijk}} \times 100$$

Therefore, the weighted row mean by number of residents is also the pooled rate of colonizations by bed size. Similarly, the weighted column mean by number of residents is also the pooled rate of colonizations by regions, given by

$$m_{+j} = \frac{\sum_i \sum_k c_{ijk}}{\sum_i \sum_k n_{ijk}} \times 100.$$

Each region j received its weighted mean rate m_{+j} (for that region), and all regions received the weighted mean rates m_{i+} (for each bed size stratum i). For benchmarking purposes, each home received the m_{+j} value for their region j and the m_{i+} value for their bed size i. We summarize the mean equations in Table 1.

$m_{11} = \frac{\sum_k c_{11k}}{\sum_k n_{11k}} \times 100$	$\cdots$	$m_{1J} = \frac{\sum_k c_{1Jk}}{\sum_k n_{1Jk}} \times 100$	$m_{1+} = \frac{\sum_j \sum_k c_{1jk}}{\sum_j \sum_k n_{1jk}} \times 100$
$\vdots$	$\ddots$	$\vdots$	$\vdots$
$m_{I1} = \frac{\sum_k c_{I1k}}{\sum_k n_{I1k}} \times 100$	$\cdots$	$m_{IJ} = \frac{\sum_k c_{IJk}}{\sum_k n_{IJk}} \times 100$	$m_{I+} = \frac{\sum_j \sum_k c_{Ijk}}{\sum_j \sum_k n_{Ijk}} \times 100$
$m_{+1} = \frac{\sum_i \sum_k c_{i1k}}{\sum_i \sum_k n_{i1k}} \times 100$	$\cdots$	$m_{+J} = \frac{\sum_i \sum_k c_{iJk}}{\sum_i \sum_k n_{iJk}} \times 100$	

Table 1: Weighted mean by number of residents. The contingency table is divided by bed sizes ($i = 1, \ldots, I$) and regions ($j = 1, \ldots J$), with multiple facilities per cell ($k = 1, \ldots, K_{ij}$).

The secure computation of these values by the Aggregator is straight forward as all of the computations are sums. The numerator and denominator are computed separately and send to the KH to decrypt these values and divide them and multiply by 100 to get the rate. The values sent by the LTCH's to the Aggregator would be:

$$[\![n_{ijk}]\!]$$
$$[\![c_{ijk}]\!]$$

Weighted Sample Variances

Similarly, we calculated the (unbiased) weighted sample variances to accompany the above means. The weighted variance for cell (i, j) is:

$$\sigma_{ij}^2 = \frac{\sum_k n_{ijk}\left(r_{ijk} - m_{ij}\right)^2}{\left(\left(\sum_k n_{ijk}\right)^2 - \left(\sum_k n_{ijk}^2\right)\right) \Big/ \sum_k n_{ijk}},$$

the weighted sample variance for row i is:

$$\sigma_{i+}^2 = \frac{\sum_j n_{ij+}\left(m_{ij} - m_{i+}\right)^2}{\left(\left(\sum_j n_{ij+}\right)^2 - \left(\sum_j n_{ij+}^2\right)\right) \Big/ \sum_j n_{ij+}},$$

and the weighted sample variance for column j is

$$\sigma_{+j}^2 = \frac{\sum_i n_{ij+}\left(m_{ij} - m_{+j}\right)^2}{\left(\left(\sum_i n_{ij+}\right)^2 - \left(\sum_i n_{ij+}^2\right)\right) \Big/ \sum_i n_{ij+}}.$$

We summarize the variance equations in Table 2.

$\sigma_{11}^2 = \frac{\sum_k n_{11k}\left(r_{11k}-m_{11}\right)^2}{\left(\frac{\left(\left(\sum_k n_{11k}\right)^2-\left(\sum_k n_{11k}^2\right)\right)}{\sum_k n_{11k}}\right)}$	$\cdots$	$\sigma_{1J}^2 = \frac{\sum_k n_{1Jk}\left(r_{1Jk}-m_{1J}\right)^2}{\left(\frac{\left(\left(\sum_k n_{1Jk}\right)^2-\left(\sum_k n_{1Jk}^2\right)\right)}{\sum_k n_{1Jk}}\right)}$	$\sigma_{1+}^2 = \frac{\sum_j n_{1j+}\left(m_{1j}-m_{1+}\right)^2}{\left(\frac{\left(\left(\sum_j n_{1j+}\right)^2-\left(\sum_j n_{1j+}^2\right)\right)}{\sum_j n_{1j+}}\right)}$
$\vdots$	$\ddots$	$\vdots$	$\vdots$
$\sigma_{I1}^2 = \frac{\sum_k n_{I1k}\left(r_{I1k}-m_{I1}\right)^2}{\left(\frac{\left(\left(\sum_k n_{I1k}\right)^2-\left(\sum_k n_{I1k}^2\right)\right)}{\sum_k n_{I1k}}\right)}$	$\cdots$	$\sigma_{IJ}^2 = \frac{\sum_k n_{IJk}\left(r_{IJk}-m_{IJ}\right)^2}{\left(\frac{\left(\left(\sum_k n_{IJk}\right)^2-\left(\sum_k n_{IJk}^2\right)\right)}{\sum_k n_{IJk}}\right)}$	$\sigma_{I+}^2 = \frac{\sum_j n_{Ij+}\left(m_{Ij}-m_{I+}\right)^2}{\left(\frac{\left(\left(\sum_j n_{Ij+}\right)^2-\left(\sum_j n_{Ij+}^2\right)\right)}{\sum_j n_{Ij+}}\right)}$
$\sigma_{+1}^2 = \frac{\sum_i n_{i1+}\left(m_{i1}-m_{+1}\right)^2}{\left(\frac{\left(\left(\sum_i n_{i1+}\right)^2-\left(\sum_i n_{i1+}^2\right)\right)}{\sum_i n_{i1+}}\right)}$	$\cdots$	$\sigma_{+J}^2 = \frac{\sum_i n_{iJ+}\left(m_{iJ}-m_{+J}\right)^2}{\left(\frac{\left(\left(\sum_i n_{iJ+}\right)^2-\left(\sum_i n_{iJ+}^2\right)\right)}{\sum_i n_{iJ+}}\right)}$	

Table 2: Weighted variance by number of residents. The contingency table is divided by bed sizes ($i = 1,\ldots,I$) and regions ($j = 1,\ldots J$), with multiple facilities per cell ($k = 1,\ldots,K_{ij}$).

Secure Computations for Variances

We focus on the computation of the variance. The variance for a cell is given by:

$$\sigma_{ij}^2 = \frac{\sum_k n_{ijk}\left(r_{ijk}-m_{ij}\right)^2}{\left(\left(\sum_k n_{ijk}\right)^2-\left(\sum_k n_{ijk}^2\right)\right)\Big/\sum_k n_{ijk}} = \frac{\left(\sum_k n_{ijk}\right)\times\left(\sum_k n_{ijk}\left(r_{ijk}-m_{ij}\right)^2\right)}{\left(\left(\sum_k n_{ijk}\right)^2-\left(\sum_k n_{ijk}^2\right)\right)}$$

We will determine the numerator and denominator separately. For the numerator the first term is easily computed. The second term can be broken down as follows:

$$\sum_k n_{ijk}\left(r_{ijk}-m_{ij}\right)^2 = n_{ij1}\left(r_{ij1}-m_{ij}\right)^2 + n_{ij2}\left(r_{ij2}-m_{ij}\right)^2 + \cdots + n_{ijK}\left(r_{ijK}-m_{ij}\right)^2$$

$$= n_{ij1}\left(r_{ij1}^2 + m_{ij}^2 - 2r_{ij1}m_{ij}\right)^2 + n_{ij2}\left(r_{ij2}^2 + m_{ij}^2 - 2r_{ij2}m_{ij}\right)^2 + \cdots + n_{ijK}\left(r_{ijK}^2 + m_{ij}^2 - 2r_{ijK}m_{ij}\right)^2$$

$$= 10^4 \times n_{ij1}\left(\frac{c_{ij1}^2}{n_{ij1}^2} + \left(\frac{\sum_k c_{ijk}}{\sum_k n_{ijk}}\right)^2 - 2\frac{c_{ij1}}{n_{ij1}}\frac{\sum_k c_{ijk}}{\sum_k n_{ijk}}\right) + 10^4 \times n_{ij2}\left(\frac{c_{ij2}^2}{n_{ij2}^2} + \left(\frac{\sum_k c_{ijk}}{\sum_k n_{ijk}}\right)^2 - 2\frac{c_{ij2}}{n_{ij2}}\frac{\sum_k c_{ijk}}{\sum_k n_{ijk}}\right) + \cdots$$
$$+ 10^4 \times n_{ijK}\left(\frac{c_{ijK}^2}{n_{ijK}^2} + \left(\frac{\sum_k c_{ijk}}{\sum_k n_{ijk}}\right)^2 - 2\frac{c_{ijK}}{n_{ijK}}\frac{\sum_k c_{ijk}}{\sum_k n_{ijk}}\right)$$

$$= 10^4 \times \left(\begin{array}{l} \left(\frac{c_{ij1}^2}{n_{ij1}} + n_{ij1}\left(\frac{\sum_k c_{ijk}}{\sum_k n_{ijk}}\right)^2 - 2c_{ij1}\frac{\sum_k c_{ijk}}{\sum_k n_{ijk}}\right) + \left(\frac{c_{ij2}^2}{n_{ij2}} + n_{ij2}\left(\frac{\sum_k c_{ijk}}{\sum_k n_{ijk}}\right)^2 - 2c_{ij2}\frac{\sum_k c_{ijk}}{\sum_k n_{ijk}}\right) + \cdots + \\ \left(\frac{c_{ijK}^2}{n_{ijK}} + n_{ijK}\left(\frac{\sum_k c_{ijk}}{\sum_k n_{ijk}}\right)^2 - 2c_{ijK}\frac{\sum_k c_{ijk}}{\sum_k n_{ijk}}\right) \end{array}\right)$$

$$=10^4 \times \left(\left(\sum_k \frac{c_{ijk}^2}{n_{ijk}} \right) + \left(\sum_k n_{ijk} \right) \left(\frac{\sum_k c_{ijk}}{\sum_k n_{ijk}} \right)^2 - 2 \left(\sum_k c_{ijk} \right) \left(\frac{\sum_k c_{ijk}}{\sum_k n_{ijk}} \right) \right)$$

$$=10^4 \times \left(\left(\sum_k \frac{c_{ijk}^2}{n_{ijk}} \right) + \left(\frac{\sum_k c_{ijk}}{\sum_k n_{ijk}} \right) \left(\left(\left(\sum_k n_{ijk} \right) \left(\frac{\sum_k c_{ijk}}{\sum_k n_{ijk}} \right) \right) - \left(2 \sum_k c_{ijk} \right) \right) \right)$$

Therefore, each site needs to compute:

$$\left[\!\left[\frac{c_{ijk}^2}{n_{ijk}} \right]\!\right]$$

and sends along with other encrypted values. Note that we need to perform scaling for the above fraction before encryption and the scale factor has to be sent along as well.

The denominator can be expressed as:

$$\left(\left(\sum_k n_{ijk}\right)^2 - \left(\sum_k n_{ijk}^2\right)\right)$$

Each site will also compute the square of the number of residents for each cell:

$$\left[\!\left[n_{ijk}^2 \right]\!\right]$$

and sends its encryption along with other encrypted values.

Therefore, the pieces of the computation would be:

$$= \frac{\left(\sum_k n_{ijk}\right) \times 10^4 \times \left[\left(\sum_k \frac{c_{ijk}^2}{n_{ijk}}\right) + \left(\frac{\sum_k c_{ijk}}{\sum_k n_{ijk}}\right)\left(\left(\sum_k n_{ijk}\left(\frac{\sum_k c_{ijk}}{\sum_k n_{ijk}}\right)\right) - \left(2\sum_k c_{ijk}\right)\right)\right]}{\left(\sum_k n_{ijk}\right)^2 - \left(\sum_k n_{ijk}^2\right)}$$

The Aggregator would therefore receive for each facility the following values:

$$\llbracket n_{ijk} \rrbracket$$
$$\llbracket c_{ijk} \rrbracket$$
$$\left\llbracket \left\lfloor \frac{c_{ijk}^2}{n_{ijk}} \times p \right\rfloor \right\rrbracket$$
$$\llbracket n_{ijk}^2 \rrbracket$$

Note that the third value, the division, is constructed to always produce an integer value: the fractional term is multiplied by a precision factor p and then truncated.

The Aggregator will compute the following values and then send them to the KH:

$$\prod_k \llbracket n_{ijk} \rrbracket = \left\llbracket \sum_k n_{ijk} \right\rrbracket$$
$$\prod_k \left\llbracket \frac{c_{ijk}^2}{n_{ijk}} \right\rrbracket = \left\llbracket \sum_k \left\lfloor \frac{c_{ijk}^2}{n_{ijk}} \times p \right\rfloor \right\rrbracket$$
$$\prod_k \llbracket c_{ijk} \rrbracket = \left\llbracket \sum_k c_{ijk} \right\rrbracket$$
$$\prod_k \llbracket n_{ijk}^2 \rrbracket = \left\llbracket \sum_k n_{ijk}^2 \right\rrbracket$$

The KH will decrypt these values as follows:

$$n_{ij+} = D\left(E\left(\sum_k n_{ijk}\right)\right)$$
$$x = \sum_k \frac{c_{ijk}^2}{n_{ijk}} = D\left(E\left(\sum_k \left\lfloor \frac{c_{ijk}^2}{n_{ijk}} \times p \right\rfloor \right)\right)$$
$$c_{ij+} = D\left(E\left(\sum_k c_{ijk}\right)\right)$$
$$y = D\left(E\left(\sum_k n_{ijk}^2\right)\right)$$

Then the KH will compute the standard deviation for a cell as follows:

$$\sqrt{\frac{n_{ij+} \times 10^4 \times \left[x + \left(\frac{c_{ij+}}{n_{ij+}} \right) \left(\left(n_{ij+} \left(\frac{c_{ij+}}{n_{ij+}} \right) \right) - \left(2c_{ij+} \right) \right) \right]}{n_{ij+}^2 - y}}$$

And for a row i we have the variance:

$$\sigma_{i+}^2 = \frac{\sum_j n_{ij+} \left(m_{ij} - m_{i+} \right)^2}{\left(\left(\sum_j n_{ij+} \right)^2 - \left(\sum_j n_{ij+}^2 \right) \right) \Big/ \sum_j n_{ij+}} = \frac{\sum_j n_{ij+} \times \sum_j n_{ij+} \left(m_{ij} - m_{i+} \right)^2}{\left(\sum_j n_{ij+} \right)^2 - \left(\sum_j n_{ij+}^2 \right)}$$

For the second term in the numerator we have:

$$\sum_j n_{ij+}\left(m_{ij}-m_{i+}\right)^2 = n_{i1+}\left(m_{i1}-m_{i+}\right)^2+\cdots+n_{iJ+}\left(m_{iJ}-m_{i+}\right)^2$$

$$= n_{i1+}\left(m_{i1}^2+m_{i+}^2-2m_{i1}m_{i+}\right)^2+\cdots+n_{iJ+}\left(m_{iJ}^2+m_{i+}^2-2m_{iJ}m_{i+}\right)^2$$

$$=10^4\times n_{i1+}\left(\left(\frac{\sum_k c_{i1k}}{\sum_k n_{i1k}}\right)^2+\left(\frac{\sum_j\sum_k c_{ijk}}{\sum_j\sum_k n_{ijk}}\right)^2-2\frac{\sum_k c_{i1k}}{\sum_k n_{i1k}}\times\frac{\sum_j\sum_k c_{ijk}}{\sum_j\sum_k n_{ijk}}\right)+\cdots+$$

$$10^4\times n_{iJ+}\left(\left(\frac{\sum_k c_{iJk}}{\sum_k n_{iJk}}\right)^2+\left(\frac{\sum_j\sum_k c_{ijk}}{\sum_j\sum_k n_{ijk}}\right)^2-2\frac{\sum_k c_{iJk}}{\sum_k n_{iJk}}\times\frac{\sum_j\sum_k c_{ijk}}{\sum_j\sum_k n_{ijk}}\right)$$

$$=10^4\times n_{i1+}\left(\left(\frac{c_{i1+}}{n_{i1+}}\right)^2+\left(\frac{c_{i++}}{n_{i++}}\right)^2-2\frac{c_{i1+}}{n_{i1+}}\times\frac{c_{i++}}{n_{i++}}\right)+\cdots+10^4\times n_{iJ+}\left(\left(\frac{c_{iJ+}}{n_{iJ+}}\right)^2+\left(\frac{c_{i++}}{n_{i++}}\right)^2-2\frac{c_{iJ+}}{n_{iJ+}}\times\frac{c_{i++}}{n_{i++}}\right)$$

$$=10^4\times\left(\frac{c_{i1+}^2}{n_{i1+}}+n_{i1+}\left(\frac{c_{i++}}{n_{i++}}\right)^2-2\frac{c_{i1+}\times c_{i++}}{n_{i++}}\right)+\cdots+10^4\times\left(\frac{c_{iJ+}^2}{n_{iJ+}}+n_{in+}\left(\frac{c_{i++}}{n_{i++}}\right)^2-2\frac{c_{iJ+}\times c_{i++}}{n_{i++}}\right)$$

$$=10^4\times\left(\sum_j\frac{c_{ij+}^2}{n_{ij+}}+\left(\frac{c_{i++}}{n_{i++}}\right)^2\sum_j n_{ij+}-2\frac{c_{i++}}{n_{i++}}\sum_j c_{ij+}\right)$$

For the denominator we have:

$$\left(\left(\sum_j n_{ij+}\right)^2 - \left(\sum_j n_{ij+}^2\right)\right) = \left(\left(n_{i++}\right)^2 - \left(\sum_j n_{ij+}^2\right)\right)$$

Therefore, each facility sends the following values to the Aggregator:

$$[\![n_{ijk}]\!]$$
$$[\![c_{ijk}]\!]$$

The Aggregator computes the following values and sends them to the KH:

$$\prod_k [\![c_{ijk}]\!] = [\![\sum_k c_{ijk}]\!]$$
$$\prod_k [\![n_{ijk}]\!] = [\![\sum_k n_{ijk}]\!]$$
$$\prod_j \prod_k [\![c_{ijk}]\!] = [\![\sum_j \sum_k c_{ijk}]\!]$$
$$\prod_j \prod_k [\![n_{ijk}]\!] = [\![\sum_j \sum_k n_{ijk}]\!]$$

The KH will decrypt these values as follows:

$$c_{ij+} = D\left(E\left(\sum_k c_{ijk}\right)\right)$$
$$n_{ij+} = D\left(E\left(\sum_k n_{ijk}\right)\right)$$
$$c_{i++} = D\left(E\left(\sum_j \sum_k c_{ijk}\right)\right)$$
$$n_{i++} = D\left(E\left(\sum_j \sum_k n_{ijk}\right)\right)$$

And then the KH performs the following computation:

$$\sigma_{i+}=\sqrt{\frac{\sum_j n_{ij+}\times 10^4\times\left(\sum_j \frac{c_{ij+}^2}{n_{ij+}}+\left(\frac{c_{i++}}{n_{i++}}\right)^2\sum_j n_{ij+}-2\frac{c_{i++}}{n_{i++}}\sum_j c_{ij+}\right)}{n_{i++}^2-\left(\sum_j n_{ij+}^2\right)}}$$

For columns the computations are similar:

$$\sum_i n_{ij+}\left(m_{ij}-m_{+j}\right)^2=n_{1j+}\left(m_{1j}-m_{+j}\right)^2+\cdots+n_{Ij+}\left(m_{Ij}-m_{+j}\right)^2$$

$$=n_{1j+}\left(m_{1j}^2+m_{+j}^2-2m_{1j}m_{+j}\right)^2+\cdots+n_{Ij+}\left(m_{Ij}^2+m_{+j}^2-2m_{Ij}m_{+j}\right)^2$$

$$=10^4\times n_{1j+}\left(\left(\frac{\sum_k c_{1jk}}{\sum_k n_{1jk}}\right)^2+\left(\frac{\sum_i\sum_k c_{ijk}}{\sum_i\sum_k n_{ijk}}\right)^2-2\frac{\sum_k c_{1jk}}{\sum_k n_{1jk}}\times\frac{\sum_i\sum_k c_{ijk}}{\sum_i\sum_k n_{ijk}}\right)+\cdots+$$

$$10^4\times n_{Ij+}\left(\left(\frac{\sum_k c_{Ijk}}{\sum_k n_{Ijk}}\right)^2+\left(\frac{\sum_i\sum_k c_{ijk}}{\sum_i\sum_k n_{ijk}}\right)^2-2\frac{\sum_k c_{Ijk}}{\sum_k n_{njk}}\times\frac{\sum_i\sum_k c_{ijk}}{\sum_i\sum_k n_{ijk}}\right)$$

$$=10^4\times n_{1j+}\left(\left(\frac{c_{1j+}}{n_{1j+}}\right)^2+\left(\frac{c_{+j+}}{n_{+j+}}\right)^2-2\frac{c_{1j+}}{n_{1j+}}\times\frac{c_{+j+}}{n_{+j+}}\right)+\cdots+10^4\times n_{Ij+}\left(\left(\frac{c_{Ij+}}{n_{Ij+}}\right)^2+\left(\frac{c_{+j+}}{n_{+j+}}\right)^2-2\frac{c_{Ij+}}{n_{Ij+}}\times\frac{c_{+j+}}{n_{+j+}}\right)$$

$$=10^4\times\left(\frac{c_{1j+}^2}{n_{1j+}}+n_{1j+}\left(\frac{c_{+j+}}{n_{+j+}}\right)^2-2\frac{c_{1j+}\times c_{+j+}}{n_{+j+}}\right)+\cdots+10^4\times\left(\frac{c_{Ij+}^2}{n_{Ij+}}+n_{Ij+}\left(\frac{c_{+j+}}{n_{+j+}}\right)^2-2\frac{c_{Ij+}\times c_{+j+}}{n_{+j+}}\right)$$

$$=10^4\times\left(\sum_i\frac{c_{ij+}^2}{n_{ij+}}+\left(\frac{c_{+j+}}{n_{+j+}}\right)^2\sum_i n_{ij+}-2\frac{c_{+j+}}{n_{+j+}}\sum_i c_{ij+}\right)$$

For denominator in the row version:

$$\left(\left(\sum_i n_{ij+}\right)^2-\left(\sum_i n_{ij+}^2\right)\right)=\left(\left(n_{+j+}\right)^2-\left(\sum_i n_{ij+}^2\right)\right)$$

Therefore. Each facility sends the following values to the Aggregator:

$$\llbracket n_{ijk} \rrbracket$$
$$\llbracket c_{ijk} \rrbracket$$

The Aggregator computes the following values and sends them to the KH:

$$\prod_k \llbracket c_{ijk} \rrbracket = \llbracket \sum_k c_{ijk} \rrbracket$$
$$\prod_k \llbracket n_{ijk} \rrbracket = \llbracket \sum_k n_{ijk} \rrbracket$$
$$\prod_i \prod_k \llbracket c_{ijk} \rrbracket = \llbracket \sum_i \sum_k c_{ijk} \rrbracket$$
$$\prod_i \prod_k \llbracket n_{ijk} \rrbracket = \llbracket \sum_i \sum_k n_{ijk} \rrbracket$$

The KH will decrypt these values as follows:

$$c_{ij+} = D\left(E\left(\sum_k c_{ijk}\right)\right)$$
$$n_{ij+} = D\left(E\left(\sum_k n_{ijk}\right)\right)$$
$$c_{+j+} = D\left(E\left(\sum_i \sum_k c_{ijk}\right)\right)$$
$$n_{+j+} = D\left(E\left(\sum_i \sum_k n_{ijk}\right)\right)$$

And then the KH performs the final calculation for the column standard deviation as follows:

$$\sqrt{\frac{\sum_i n_{ij+} \times 10^4 \times \left(\sum_i \frac{c_{ij+}^2}{n_{ij+}} + \left(\frac{c_{+j+}}{n_{+j+}} \right)^2 \sum_i n_{ij+} - 2\frac{c_{+j+}}{n_{+j+}} \sum_i c_{ij+} \right)}{n_{+j+}^2 - \left(\sum_i n_{ij+}^2 \right)}}$$

Security Analysis

In this section we analyze the security of the data collection system in two settings. The first setting we define an ideal functionality and prove our protocol implements this functionality in the semi-trusted model. The standard goal for such an analysis is to show that the parties learn nothing more about each other's private inputs than what is revealed by the output itself. In the second setting we analyze what an adversary can learn about the private inputs from the output.

We specifically consider the case of the information provided by the LTCH's to compute the cell standard deviation because that is a superset the information that is provided for the other statistics. A security proof for the more detailed information for the cell standard deviation would also apply to any subset of information.

Setting 1 — Security of Private Data Collection Protocol

Loosely speaking, a protocol is secure in the semi-trusted adversarial model if a party learns nothing more from participating in the protocol than it learns from the inputs and outputs available to that party.

We proceed by defining an ideal private data collection protocol in the presence of a trusted third party (TTP). We then review our semi-trusted private data collection protocol described earlier, and proceed to show its participants learn nothing more from the execution of this protocol than they would in the ideal implementation, and in turn, that our private data collection protocol is secure against a semi-trusted adversary.

Ideal Private Data Collection

Private data collection (PDC) is ideally implemented between K long-term care homes $H_1 \ldots H_K$, an Aggregator and key holder KH as follows: each home H_i sends to TTP the number of residents n_i, and the number of cases c_i of cases. The TTP then sends to KH the following:

$$sn = \sum_{i=1}^{k} n_i$$

$$sc = \sum_{i=1}^{k} c_i$$

$$sscn = \sum_{i=1}^{k} \left\lfloor \frac{c_i^2}{n_i} \times p \right\rfloor$$

$$ssn = \sum_{i=1}^{k} n_i^2 .$$

Then $H_1 \ldots H_K$ and the Aggregator receive no output.

Semi-trusted Private Data Collection Protocol

The semi-trusted private data collection protocol (PDC-semi-trusted) is implemented between K LTCH's $H_1 \ldots H_k$, Aggregator and key holder KH. KH generates a public and private key pair, and broadcasts the public key to all parties. Each home H_i then sends to the Aggregator:

$$[\![n_i]\!]$$

$$[\![c_i]\!]$$

$$\left[\!\!\left[\left\lfloor \frac{c_i^2}{n_i} \cdot p \right\rfloor\right]\!\!\right]$$

$$[\![n_i^2]\!].$$

The Aggregator homomorphically computes the following values and sends them to KH:

$$[\![sn]\!] = \left[\!\!\left[\sum_{i=1}^{k} n_i\right]\!\!\right] = \prod_{i=1}^{k} [\![n_i]\!]$$

$$[\![sc]\!] = \left[\!\!\left[\sum_{i=1}^{k} c_i\right]\!\!\right] = \prod_{i=1}^{k} [\![c_i]\!]$$

$$[\![sscn]\!] = \left[\!\!\left[\sum_{i=1}^{k} \left\lfloor \frac{c_i^2}{n_i} \cdot p \right\rfloor\right]\!\!\right] = \prod_{i=1}^{k} \left[\!\!\left[\left\lfloor \frac{c_i^2}{n_i} \cdot p \right\rfloor\right]\!\!\right]$$

$$[\![ssn]\!] = \left[\!\!\left[\sum_{i=1}^{k} n_i^2\right]\!\!\right] = \prod_{i=1}^{k} [\![n_i^2]\!].$$

KH recovers $sn, sc, sscn,$ and ssn by decrypting the ciphertexts it received from the Aggregator.

Security of Protocol PDC-Semi-Trusted

We state the claims of security for PDC in the semi-trusted model.

Lemma 1 (Correctness). *Protocol PDC-semi-trusted evaluates the PDC functionality with high probability.*

The proof is based on the fact that KH receives encryptions of $sn, sc, sscn,$ and ssn.

Lemma 2 (Privacy is preserved between homes). *For each home H_i, every other home $H_{j \neq i}$ learns nothing about H_i's input.*

The proof is based on the fact that homes receive no information from the protocol execution.

Lemma 3 (Privacy is preserved between homes and the Aggregator). *If the encryption scheme is semantically secure, then the protocol views of the Aggregator for any two inputs of any home H_i are indistinguishable.*

The proof is based on the fact that the only information the Aggregator receives consists of semantically-secure encryptions and does not know the associated private key.

Lemma 4 (Privacy is preserved between homes and KH). *KH learns nothing more about each home H_i than it does from $sn, sc, sscn,$ and ssn.*

The proof is based on the fact that KH only receives the encryptions of $sn, sc, sscn,$ and ssn.

Theorem 1 (PDC-semi-trusted is secure in the semi-trusted model). *Each participant learns nothing more from an execution of PDC-semi-trusted that it does from an execution of PDC on the same inputs.*

The proof follows from lemmas 1-4.□

Setting 2 — Anonymity of Values

Now that we have discussed the security of PDC-semi-trusted**,** we turn our attention to an important question: what can KH learn about the colonization rate of individual homes given the output? Recall that the purpose of PDC-semi-trusted is to provide homes with a means of contributing data to public health statistics without having to reveal its individual colonization rate. If these figures can be reverse engineered from the output, the private data collection system cannot be considered secure, even if the protocol itself is.

In particular we consider the question of how much an adversary can infer about each individual home H_i's colonization rate $\frac{c_i}{n_i}$ given the values $sn, sc, sscn, ssn, K$ and p.

Definitions

Definition (ground truth): Let $S = \{N, C\}$ respectively be the actual resident numbers $N = \{n_1, \ldots, n_k\}$ and case counts $C = \{c_1, \ldots, c_k\}$ for a particular collection of K LTCHs. Let $R = \{sn, sc, sscn, ssn\}$ be defined by the following system of equations:

$$sn = \sum_{i=1}^{k} n_i$$

$$sc = \sum_{i=1}^{k} c_i$$

$$sscn = \sum_{i=1}^{k} \left\lfloor \frac{c_i^2}{n_i} \cdot p \right\rfloor$$

$$ssn = \sum_{i=1}^{k} n_i^2.$$

We call R the *result* of the above system of equations evaluated under S. We say S is the *ground truth* of R.

Definition (solutions of R): Let $R = \{sn, sc, sscn, ssn\}$. Let $S' = \{N', C'\}$ for $N' = \{n_1', \ldots, n_k'\}$ and $C' = \{c_1', \ldots, c_k'\}$. We call S' a *solution* of R if:

$$sn = \sum_{i=1}^{k} n_i'$$

$$sc = \sum_{i=1}^{k} c_i'$$

$$sscn = \sum_{i=1}^{k} \left\lfloor \frac{c_i^{2\,\prime}}{n_i'} \times p \right\rfloor$$

$$ssn = \sum_{i=1}^{k} n_i^{2\,\prime}.$$

Let S be the ground truth of R. We call any solution $S' \neq S$ a *spurious solution* of R.

Definition (identifying result): Let R be the result of the ground truth solution S. Let $S_1, \ldots, S_\ell$ be the ℓ spurious solutions of R. We call $\widehat{S_R} = \{S_1, \ldots, S_\ell, S\}$ the *solution set* of R. We say that result R is t-*identifying* if $|\widehat{S_R}| < t$ for some threshold t. We say that R is t-*non-identifying* if $|\widehat{S_R}| \geq t$.

Definition (anonymity): Let $\hat{R}$ be an ensemble of results vectors. Let p_{ID} be the minimally acceptable probability that a particular result R is t-non-identifying. We say the results ensemble $\hat{R}$ is $anonymous$ if:

$$\forall R \in \hat{R}\colon \mathbf{Pr}\left[|\widehat{S_R}| \geq t\right] \geq p_{ID}.$$

In other words, the ensemble of results $\hat{R}$ is anonymous if each result $R \in \hat{R}$ is t-non-identifying with probability greater than or equal to p_{ID}.

Definition (integer partition): Let n be an integer. An *integer partition* of n is defined as any vector of integers $\{n_1, \ldots, n_q\}$ for which $\sum_{i=1}^{q} n_i = n$ and arbitrary q. We define a k-partition of n as any vector of integers $\{n_1 \ldots n_k\}$ for which $\sum_{i=1}^{k} n_i = n$.

Concrete example of a result with multiple solutions.

Suppose we have $k = 6$ sites. Assume it is publicly known that each home houses $60 - 120$ patients, and that the average colonization rate across all sites is 5%. Consider the following scenario in which the ground truth $S = \{N, C\}$ is as follows:

i	1	2	3	4	5	6
n_i	115	112	96	90	80	66
c_i	0	6	6	2	6	8

For compactness, we write this as:

$$S = \{\{115, 112, 96, 90, 80, 66\}, \{0, 6, 6, 2, 6, 8\}\}.$$

Using a precision of $p = 10^6$, S yields the following result:

$$sn = \sum_{i=1}^{k} n_i = 559$$

$$sc = \sum_{i=1}^{k} c_i = 28$$

$$sscn = \sum_{i=1}^{k} \left\lfloor \frac{c_i^2}{n_i} \cdot p \right\rfloor = 2160568$$

$$ssn = \sum_{i=1}^{k} n^2 = 53841,$$

$i.e., R = \{559, 28, 2160568, 53841\}$. We wrote a software tool to search for all possible solutions given Runder the assumptions above and found $|\widehat{S}| = 100$ solutions: 1 solution corresponding to the ground truth, and 99 spurious solutions. Because $|\widehat{S}| \geq 5$ we say R is non-identifying.

As a concrete demonstration of the software's ability to compute solution set $|\widehat{S}|$, we list a few of the spurious solutions below:

$$S_1 = \{\{120, 112, 90, 86, 76, 75\}, \{4, 12, 0, 7, 3, 2\}\},$$

$$S_2 = \{\{118, 111, 93, 91, 75, 71\}, \{2, 1, 6, 4, 7, 8\}\},$$

$$S_3 = \{\{115, 112, 102, 80, 78, 72\}, \{2, 3, 4, 9, 8, 2\}\},$$

$$S_4 = \{\{111, 110, 106, 88, 78, 66\}, \{4, 5, 1, 10, 7, 1\}\},$$

$$S_5 = \{\{107, 104, 104, 100, 86, 58\}, \{0, 5, 9, 5, 2, 7\}\},$$

$$S_6 = \{\{109, 105, 101, 94, 93, 57\}, \{9, 3, 3, 6, 0, 7\}\}.$$

Note that all of these solutions will satisfy the above system of equations for the example.

Simulating the anonymity of results.

We wrote a software tool to estimate whether a particular results ensemble was anonymous or not. For the purposes of this study, we define a results ensemble in terms of two variables: the number of sites

k, and the lower and upper bounds on the number of patients at each site $b_\ell \leq n \leq b_u$. Consistent with the size of the LTCHs in our actual study, we set $b_u \in \{60,120,180\}$. As a simplifying assumption, we fixed $b_\ell = 0.5 \cdot b_u$. We computed values for $k \in \{5,6,7,8,9,10\}$ sites. As in the actual study, we set precision $p = 10^6$.

For each parameterization $\{k, b_u\}$, we ran multiple replicates of the following simulation:

1. Randomly generate patient count vector $N = \{n_1, \dots, n_k\}$ where $b_\ell \leq n_i \leq b_u$. Compute sn and ssn from the equations above.
2. Set case count to be $sc = sn \cdot r$ for a set average colonization rate r. For our study we considered the rates $r \in \{0.01, 0.03, 0.05\}$. Randomly generate the case count vector $C = \{c_1, \dots, c_k\}$ by computing all k-partitions of sc and selecting one at random. Compute $sscn$ using N and C from the equations above. $S = \{N, C\}$ represents the ground truth. The result is $R = \{sn, ssn, sc, sscn\}$
3. Compute the cardinality of solution set $|\widehat{S_R}|$ of R as follows:
   a. Compute all possible candidates for N by computing all vectors $\{n_1, \dots, n_k\}$ for which $\sum_{i=1}^{k} n = sn, \sum_{i=1}^{k} n^2 = ssn$ and for which $b_\ell \leq n_i \leq b_u$.
   b. Compute all possible candidates for C by computing for all k-partitions of sc.
   c. For all candidate N's and all valid shuffling of all candidate C's, compute the resulting candidate $sscn$ from the equations above. If this value is equal to the actual $sscn$, increment a counter representing $|\widehat{S_R}|$.
4. Output the value of the $|\widehat{S_R}|$ counter.

Simulation results.

Using the data collected from the simulations, we constructed a model to consider when the combination of (b_u, k, r) is 5-identifying, i.e., $|\widehat{S_R}| < 5$. The outcome was made binary: 0 if $|\widehat{S_R}| \geq 5$ (non-identifying); 1 if $|\widehat{S_R}| < 5$ (identifying). There were 13,034 observations, 4,350 of which were identifying cases. Multicollinearity was not detected—the variance inflation factor was less than 2 for the main effects, (b_u, k, r).

We started with a main effects model, which fit better than the null model, and sufficiently well compared to the saturated model, according to likelihood-ratio tests. The unweighted sum of squares goodness-of-fit test[18], however, detected a lack of fit for the main effects model. Only when we included the 3-way interaction did the global goodness-of-fit test fail to detect a lack of fit, with a p-value of 0.202. Adding the 3-way interaction also produced a statistically significant decrease in the residual deviance, with a value of 5,703 for the final model. The resulting model behaves as we expect. We provide a summary in the following table.

**Coefficient**	**Log Odds**	**95% CI**	**p-value**
Intercept	5.464	(1.283, -9.646)	0.010
Number of Sites (k)	-2.098	(-2.851, -1.346)	0.000
Number of Patients (b_u)	0.023	(-0.020, 0.067)	0.296
Colonization Rate (r)	-0.954	(-2.752, 0.843)	0.298
2-way interaction $k * b_u$	0.005	(-0.003, 0.012)	0.245
2-way interaction $k * r$	0.679	(0.369, 0.989)	0.000
2-way interaction $b_u * r$	0.056	(0.036, 0.076)	0.000
3-way interaction $k * b_u * r$	-0.014	(-0.017, -0.011	0.000

Table 3: Logistic regression model of 5-identifiability.

Given the high probability of 5-identifiability for 60-patient facilities with high colonization rates, based on our model, we decided to predict at higher rates of colonization than was originally simulated. Note that our model had a low predictive error rate of only 0.098, compared to the null model's error rate of 0.334, and provided a high-level of predictive discrimination with a c-index of 0.964.

The following figure shows the predicted probability of 5-identifiability for colonization rates ranging from 7 to 10%. When there are twelve 60-patient facilities, the upper confidence limit is below 0.05 for a colonization rate of 10% or less; when there are eleven 60-patient facilities, the upper confidence limit is below 0.05 for a colonization rate of 8% or less.

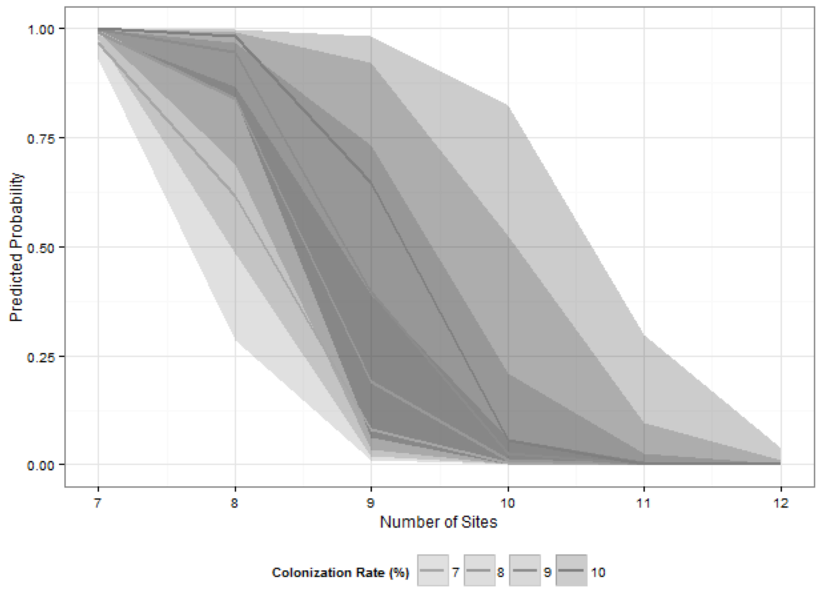

Figure 1: Predicting 5-identifying combinations, for the worst case of 60-patient facilities. Lines represent the mean; shaded areas represent the 95% confidence intervals.

If we compare to facilities of different capacities, however, we find that far fewer sites are needed to ensure the probability of 5-identifiability is less than 0.05 for a colonization rate of 10% or less. We summarize the minimum number of facilities required in the following table.

**Number of Patients** (b_u)	**Number of Sites** (k)
60	12
120	7
180	6
240	6

Table 4: Number of sites needed to ensure Pr(5-identifiability) ≤ 0.05 for a10% colonization rate.

The case with no standard deviations per cell

In certain circumstances we may not wish to calculate the standard deviation in a particular cell. In this case the participants can skip decrypting the values $sscn$ and ssn. It should be obvious that if $R =$

$\{sn, sc, sscn, ssn\}$ is t-non-identifying, then the system of equations defined by a subset of R should be as well. For completeness, we offer the following proof.

Definition (solutions of result subset R_{SUB}): Let $R_{SUB} = \{sn, sc\}$. Let $S_{SUB}' = \{N', C'\}$ for some $N' = \{n_1', \dots, n_k'\}$ and $C' = \{c_1', \dots, c_k'\}$. We call S_{SUB}' a *solution* of R_{SUB} if:

$$sn = \sum_{i=1}^{k} n_i'$$

$$sc = \sum_{i=1}^{k} c_i'$$

Theorem (anonymity of R_{SUB}): If result R is t-non-identifying then result subset R_{SUB} is also t-non-identifying.

Proof: The intuition of the proof is straightforward: R_{SUB} contains less information than R, and thus cannot ever provide us with *more* help in deducing the ground truth. Therefore if the ground truth is sufficiently obscured by R then so to is it by R_{SUB}.

Assume R is t-non-identifying, i.e., the cardinality of the set of possible solutions $\widehat{S_R}$ is greater than or equal to t. Since $R_{SUB} \subset R$ then $\widehat{S_{R_{SUB}}} \supseteq \widehat{S_R}$, and hence $|\widehat{S_{R_{SUB}}}| \geq |\widehat{S_R}| \geq t$. In other words, because R_{SUB} is a subset of R, then whatever solution is contained in $\widehat{S_R}$ is also contained in $\widehat{S_{R_{SUB}}}$. Therefore $|\widehat{S_{R_{SUB}}}|$ is at least as large as $\widehat{S_R}$ and therefore is t-non-identifying. □

References

1. El Emam K, Hu J, Mercer J, Peyton L, Kantarcioglu M, et al. (2011) A Secure Protocol for Protecting the Identity of Providers When Disclosing Data for Disease Surveillance. Journal of the American Medical Informatics Association 18: 212–217.

2. El Emam K, Hu J, Samet S, Peyton L, Earle C, et al. (2012) A Protocol for the Secure Linking of Registries for HPV Surveillance. PLoS ONE 7.

3. El Emam K, Samet S, Arbuckle L, Tamblyn R, Earle C, et al. (2012) A secure distributed logistic regression protocol for the detection of rare adverse drug events. Journal of the American Medical Informatics Association. Available: http://jamia.bmj.com/content/early/2012/08/06/amiajnl-2011-000735. Accessed 5 October 2012.

4. Dolan A (2004) Social engineering. SANS Institute.

5. Winkler I, Dealy B (1995) Information security technology ? .. Don’t rely on it: A case study in social engineering. Proceedings of the Fifth USENIX UNIX Security Symposium.

6. Mitnick K, Simon W (2002) The Art of Deception: Controlling the Human Element of Security. Wiley.

7. Long J (2008) No Tech Hacking: A Guide to Social Engineering, Dumpster Diving, and Shoulder Surfing. Burlington, MA: Syngress Publishing Inc.

8. Verizon Business Risk Team (2009) 2009 data breach investigations report.

9. National Institutes of Health (n.d.) Certificates of Confidentiality Kiosk. Available: http://grants.nih.gov/grants/policy/coc/.

10. Beskow LM, Dame L, Costello EJ (2008) Certificates of Confidentiality and the Compelled Disclosure of Research Data. Science 322: 1054–1055. doi:10.1126/science.1164100.

11. Currie PM (2005) Balancing Privacy Protections with Efficient Research: Institutional Review Boards and the Use of Certificates of Confidentiality. IRB: Ethics and Human Research 27: 7. doi:10.2307/3564529.

12. Wolf L, Zandecki J (n.d.) Sleeping Better at Night: Investigators' Experiences with Certificates of Confidentiality. IRB: Ethics and Human research 28: 1–7.

13. Wolf LE, Dame LA, Patel MJ, Williams BA, Austin JA, et al. (2012) Certificates of confidentiality: legal counsels' experiences with and perspectives on legal demands for research data. J Empir Res Hum Res Ethics 7: 1–9. doi:10.1525/jer.2012.7.4.1.

14. Panel on Research Ethics (2009) Tri-Council Policy Statement: Ethical Conduct for Research Involving Humans, 2nd edition.

15. Gibson E (n.d.) Is There a Privacy Interest in Anonymised Personal Health Information?". Health Law Journal 11: 97–112.

16. Rothstein, Mark (2010) Is Deidentification Sufficient to Protect Health Privacy in Research. The American Journal of Bioethics 10: 3–11.

17. Paillier P (1999) Public-key cryptosystems based on composite degree residuosity classes. Proceedings of the 17th international conference on Theory and application of cryptographic techniques. EUROCRYPT'99. Berlin, Heidelberg: Springer-Verlag. pp. 223–238. Available: http://dl.acm.org/citation.cfm?id=1756123.1756146. Accessed 28 April 2012.

18. Hosmer DW, Hosmer T, Le Cessie S, Lemeshow S (1997) A Comparison of Goodness-of-Fit Tests for the Logistic Regression Model. Statistics in Medicine 16: 965–980. doi:10.1002/(SICI)1097-0258(19970515)16:9<965::AID-SIM509>3.0.CO;2-O.
